# Comparative outcomes of natural orifice specimen extraction surgery versus conventional minilaparotomy-assisted specimen extraction in colorectal cancer: A single-center retrospective study

**DOI:** 10.1097/MD.0000000000051179

**Published:** 2026-10-16

**Authors:** Yongjian Liao, Runfeng Qiu, Wenli Ma

**Affiliations:** a Department of Colorectal Surgery, The First People’s Hospital of Lin’an District, Hangzhou, Zhejiang, China.

**Keywords:** colorectal cancer, laparoscopy, minimally invasive surgery, natural orifice specimen extraction, patient outcomes, retrospective study

## Abstract

Natural orifice specimen extraction surgery (NOSES) has emerged as an advanced minimally invasive technique that eliminates abdominal wall incisions for specimen retrieval; nevertheless, comparative data on its efficacy relative to conventional minilaparotomy-assisted extraction in colorectal cancer (CRC) remain limited. This retrospective analysis was conducted on 126 patients who underwent laparoscopic CRC resection at our institution, allocated to either the NOSES group (transanal specimen extraction, n = 49) or the conventional group (minilaparotomy-assisted extraction, n = 77), comparing perioperative parameters, postoperative recovery indicators, complication rates, and pathological outcomes. The NOSES group demonstrated significantly shorter time to first flatus (2.3 ± 0.9 vs 2.9 ± 1.1 days, *P* = .002), time to first liquid diet (3.1 ± 1.0 vs 3.8 ± 1.3 days, *P* = .001), and postoperative hospital stay (8.4 ± 3.2 vs 10.1 ± 4.1 days, *P* = .014). Visual analog scale pain scores on postoperative day 1 were significantly lower in the NOSES group (3.2 ± 1.1 vs 4.5 ± 1.3, *P* < .001), with reduced additional analgesic requirements (22.4% vs 44.2%, *P* = .014). Overall complication rates were significantly lower in the NOSES group (12.2% vs 24.7%, *P* = .043), with wound infections occurring exclusively in the conventional group (0% vs 6.5%, *P* = .045). No significant differences were observed in operative time, estimated blood loss, anastomotic leakage, lymph node yield, or R0 resection rate (all *P* > .05). In conclusion, NOSES appears to be a safe and feasible alternative to conventional minilaparotomy-assisted specimen extraction in highly selected patients with CRC, offering advantages in postoperative pain control, gastrointestinal function recovery, and wound-related complication reduction without compromising oncological integrity.

## 1. Introduction

Colorectal cancer (CRC) constitutes a substantial global health burden, ranking as the third most prevalent malignancy and the second leading cause of cancer-related mortality worldwide.^[1]^ According to Global Cancer Statistics, approximately 1.8 million new cases and 860,000 deaths are attributed to this disease annually, with projections indicating a 60% increase in incidence by 2030.^[2,3]^ Surgical resection remains the cornerstone of curative treatment for localized CRC.

Since Jacobs et al introduced laparoscopic colorectal resection in 1991, minimally invasive approaches have progressively supplanted open surgery as the standard of care.^[4]^ A substantial body of evidence has established that laparoscopic techniques confer comparable oncological outcomes while significantly reducing postoperative pain, shortening hospital stays, and accelerating functional recovery.^[5,6]^ Nevertheless, the widespread adoption of advanced surgical platforms, such as robotic systems, warrants critical evaluation to determine whether technological escalation consistently translates into measurable patient-centered benefits, rather than being justified by novelty alone.^[7]^

Despite these well-documented advantages, conventional laparoscopic procedures still necessitate an auxiliary abdominal incision – typically 4 to 8 cm in length – for specimen retrieval.^[8]^ This requisite minilaparotomy is associated with a spectrum of wound-related complications, including surgical site infection, wound dehiscence, and incisional hernia, while the resultant abdominal scar may impose a considerable psychological burden, particularly on younger patients.^[9]^

Natural orifice specimen extraction surgery (NOSES) has emerged as an innovative strategy to circumvent these limitations. In NOSES, the resected specimen is retrieved through a natural orifice – most commonly the anus or vagina – following complete intracorporeal resection and digestive tract reconstruction, thereby obviating the need for an auxiliary abdominal incision.^[10]^ This technique theoretically preserves abdominal wall integrity and may reduce incision-related morbidity.^[11]^ The technical specifications and theoretical framework for NOSES have been systematically codified in international consensus guidelines.

Multiple meta-analyses have demonstrated that NOSES is associated with reduced postoperative pain, accelerated gastrointestinal function recovery, shorter hospital stays, and superior cosmetic outcomes relative to conventional laparoscopy.^[12,13]^ Notably, recent evidence suggests a favorable inflammatory profile, with significantly lower postoperative levels of tumor necrosis factor-α and C-reactive protein.^[14]^ Furthermore, accumulating data indicate that NOSES does not compromise oncological principles, with comparable lymph node yields, resection margins, and long-term survival rates.^[15]^

Nevertheless, the assertion that comparative data remain scarce is only partially accurate, as several meta-analyses and comparative series have already been published. Critically, the majority of existing evidence derives from heterogeneous cohorts that pool various NOSES subtypes and colorectal subsites. In this context, the originality of the present study lies in its focused, single-center analysis of a well-defined Chinese cohort, specifically restricted to transanal NOSES (Type IV) versus conventional minilaparotomy-assisted extraction in sigmoid and rectal cancer patients, with an emphasis on short-term perioperative and pathological surrogate outcomes.

Accordingly, this retrospective study was undertaken to provide a granular comparative assessment of perioperative outcomes, postoperative recovery parameters, complication profiles, and pathological results between transanal NOSES and conventional minilaparotomy-assisted specimen extraction in a homogeneous cohort of patients undergoing laparoscopic resection for sigmoid or rectal cancer at a single Chinese institution.

## 2. Materials and methods

### 2.1. Study design and patient population

This single-center retrospective cohort study included consecutive patients who underwent laparoscopic radical resection for sigmoid colon or rectal cancer at our institution between January 2021 and June 2023. Given the safety and feasibility considerations, the following selection criteria were applied, and the results should be interpreted within this context. All surgical procedures were performed in accordance with the Declaration of Helsinki. Written informed consent for the surgical procedure was obtained from all patients prior to surgery in accordance with standard clinical practice. The retrospective study protocol, including the review and analysis of anonymized medical records, was approved by the institutional ethics committee (approval number: 2023-036) on July 28, 2023. The ethics committee waived the requirement for additional informed consent for study participation or data use, given the retrospective nature of the study and the use of fully de-identified data.

Inclusion criteria were: histologically confirmed colorectal adenocarcinoma; tumor located in the sigmoid colon or rectum; age 18 to 80 years; American Society of Anesthesiologists (ASA) class ≤ III; body mass index (BMI) < 30 kg/m^2^; and maximal tumor diameter ≤5 cm on preoperative imaging.

Exclusion criteria were: distant metastasis (M1 stage); emergency surgery for obstruction, perforation, or bleeding; previous abdominal surgery precluding safe NOSES; preoperative neoadjuvant chemoradiotherapy; synchronous malignancies; inflammatory bowel disease; and incomplete clinical data.

Patients were assigned to either the NOSES group (transanal specimen extraction) or the conventional group (minilaparotomy-assisted extraction) based on the surgical approach received; no matching was performed. Of the 187 consecutive patients initially screened, 61 were excluded: 15 for emergency surgery, 13 for distant metastasis, 9 for preoperative neoadjuvant chemoradiotherapy, and 24 for incomplete clinical data. The remaining 126 patients constituted the final analytic cohort, with 49 in the NOSES group and 77 in the conventional group (Fig. 1).

**Figure 1.**
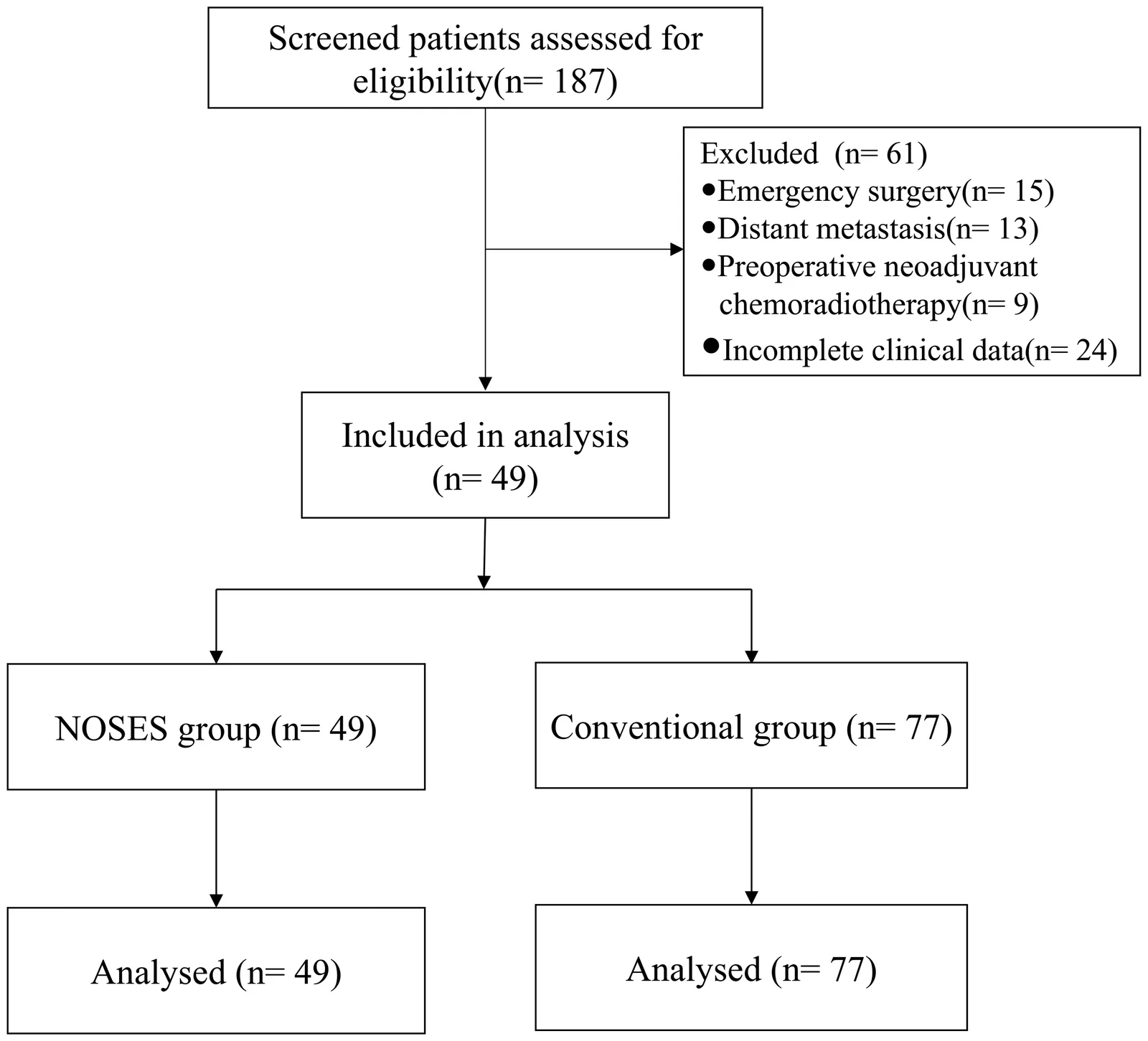
STROBE-style flow diagram of patient selection. A total of 187 consecutive patients who underwent laparoscopic radical resection for colorectal cancer were screened for eligibility. Of these, 61 patients were excluded: 15 underwent emergency surgery, 13 had distant metastasis, 9 received preoperative neoadjuvant chemoradiotherapy, and 24 had incomplete clinical data. The remaining 126 patients were included in the final analysis, with 49 patients in the NOSES group and 77 patients in the conventional group. NOSES = natural orifice specimen extraction surgery.

To minimize potential bias, all operations were performed by the same surgical team, and data were independently extracted from electronic medical records by 2 investigators with subsequent cross-verification. Blinding of outcome assessors was not applicable given the retrospective design. All eligible patients during the study period were included; no a priori sample size calculation was performed.

### 2.2. Surgical procedures

All operations were performed by the same surgical team with extensive experience in both laparoscopic colorectal surgery and NOSES techniques.

#### 2.2.1. Conventional group

Following standard laparoscopic mobilization of the colon or rectum, mesenteric vessel ligation, and total mesorectal excision (for rectal cancer) or complete mesocolic excision (for colon cancer), a 4 to 6 cm minilaparotomy incision was made in the lower abdomen. A wound protector was inserted, and the specimen was extracted through this incision. Extracorporeal resection of the bowel and placement of the anvil were performed, followed by restoration of intestinal continuity under direct vision or laparoscopic assistance.

#### 2.2.2. NOSES group

The laparoscopic resection and lymphadenectomy were performed identically to the conventional approach. For transanal specimen extraction (NOSES Type IV for sigmoid or upper rectal tumors), the rectum was transected using a linear stapler approximately 4 cm distal to the tumor. A sterile protective sleeve was introduced transanally and positioned in the rectal stump. The specimen was placed into the protective sleeve and gently extracted through the anus. The anvil of the circular stapler was then introduced transanally and secured in the proximal colon with a purse-string suture. Colorectal end-to-end anastomosis was completed using a circular stapler. The integrity of the anastomosis was verified by air leak testing, and the anastomosis was reinforced with interrupted sutures when indicated.

### 2.3. Data collection

The following data were extracted from electronic medical records:

#### 2.3.1. Demographic and baseline characteristics

Age, sex, BMI, ASA class, tumor location, preoperative carcinoembryonic antigen level.

#### 2.3.2. Perioperative parameters

Operative time, estimated blood loss, total length of all abdominal incisions (including all trocar ports and the specimen extraction incision, if applicable), conversion to open surgery.

#### 2.3.3. Postoperative recovery indicators

Time to first flatus (days), time to first liquid diet (days), time to ambulation (days), and postoperative hospital stay (days).

#### 2.3.4. Pain assessment

Visual analog scale (VAS) pain scores on postoperative days (PODs) 1, 3, and 5. Additional analgesic requirements were recorded.

#### 2.3.5. Postoperative complications

Complications were graded according to the Clavien-Dindo classification. Specific complications recorded included anastomotic leakage, wound infection, intra-abdominal abscess, ileus, urinary retention, and pulmonary infection.

#### 2.3.6. Pathological outcomes

Tumor diameter, histological differentiation, pathological T and N stages, number of harvested lymph nodes, number of positive lymph nodes, and resection margin status.

Long-term follow-up data were not available for this cohort; outcome assessment was limited to the postoperative hospital stay period.

### 2.4. Statistical analysis

Continuous variables are presented as mean ± standard deviation or median with interquartile range, as appropriate, and were compared using the independent samples *t* test or Mann–Whitney *U* test. Categorical variables are presented as frequencies and percentages and were compared using the χ^2^ test or Fisher exact test. To assess baseline comparability beyond traditional *P* values, standardized differences were calculated for all baseline covariates, with values <0.1 considered indicative of satisfactory balance. Given the limited sample size and the relatively low number of outcome events, which precluded stable multivariable modeling (overfitting risk), we did not perform propensity score matching or multivariate regression adjustment. Sensitivity analyses focusing on key clinical subgroups were performed to assess the consistency of the primary findings.

## 3. Results

### 3.1. Patient characteristics

As this was a descriptive comparative study without multivariable adjustment, only unadjusted estimates are presented. Patients with incomplete clinical data were excluded from the analysis; therefore, the final dataset contained no missing values for the variables of interest. A total of 126 patients were included in this study, comprising 49 patients in the NOSES group and 77 patients in the conventional group. The baseline characteristics of the 2 groups are summarized in Table 1. No statistically significant differences were observed between groups regarding age, sex, BMI, ASA class, tumor location, or preoperative carcinoembryonic antigen level (all *P* > .05). Notably, the standardized differences for all baseline covariates ranged from 0.04 to 0.08, all below the prespecified threshold of 0.1, indicating satisfactory covariate balance between the 2 groups and mitigating concerns regarding selection bias.

**Table 1:** Baseline characteristics of patients before propensity score matching.

Variable	NOSES group (n = 49)	Conventional group (n = 77)	*P* value	Standardized difference
Age (yr), mean ± SD	58.4 ± 10.2	60.1 ± 11.3	.386	0.07
Sex, n (%)			.492	0.06
Male	29 (59.2)	42 (54.5)		
Female	20 (40.8)	35 (45.5)		
Body mass index (kg/m^2^), mean ± SD	23.1 ± 2.7	23.8 ± 2.9	.175	0.08
ASA class, n (%)			.634	0.05
I	12 (24.5)	15 (19.5)		
II	29 (59.2)	50 (64.9)		
III	8 (16.3)	12 (15.6)		
Tumor location, n (%)			.297	0.07
Sigmoid colon	29 (59.2)	39 (50.6)		
Rectum	20 (40.8)	38 (49.4)		
Tumor diameter (cm), mean ± SD	3.2 ± 1.1	3.4 ± 1.2	.342	0.06
Preoperative CEA (ng/mL), median (IQR)	2.8 (1.6–5.2)	3.1 (1.8–5.8)	.408	0.04
Pathological stage, n (%)			.516	0.06
Stage I	13 (26.5)	18 (23.4)		
Stage II	21 (42.9)	30 (39.0)		
Stage III	15 (30.6)	29 (37.7)		

Data are presented as mean ± SD, median (IQR), or number (percentage), as appropriate. *P* values were derived from independent samples *t* test, Mann–Whitney *U* test, χ^2^ test, or Fisher exact test, as appropriate. Standardized differences <0.1 indicate satisfactory balance of baseline covariates between groups; values ≤0.1 are conventionally considered indicative of adequate comparability. This metric is less sensitive to sample size than *P* values and thus provides a more robust assessment of covariate balance.

ASA = American Society of Anesthesiologists, CEA = carcinoembryonic antigen, IQR = interquartile range, NOSES = natural orifice specimen extraction surgery, SD = standard deviation.

### 3.2. Perioperative outcomes

Perioperative parameters are presented in Table 2. The operative time was comparable between the NOSES and conventional groups (186.4 ± 42.7 minutes vs 178.9 ± 38.5 minutes, *P* = .302). Similarly, estimated blood loss did not differ significantly between the 2 groups (58.3 ± 31.6 mL vs 63.7 ± 35.2 mL, *P* = .384). Notably, the length of abdominal incision was significantly shorter in the NOSES group (4.9 ± 0.8 cm vs 11.3 ± 1.2 cm, *P* < .001; Fig. 2).

**Table 2:** Perioperative outcomes.

Parameter	NOSES group (n = 49)	Conventional group (n = 77)	*P* value
Operative time (min)	186.4 ± 42.7	178.9 ± 38.5	.302
Estimated blood loss (mL)	58.3 ± 31.6	63.7 ± 35.2	.384
Length of abdominal incision (cm)	4.9 ± 0.8	11.3 ± 1.2	<.001

In both groups, the total length of all abdominal incisions (including trocar ports and the specimen extraction incision, when present) is reported. In the conventional group, this total comprises the 5 laparoscopic trocar incisions plus the 4 to 6 cm minilaparotomy extraction incision.

NOSES = natural orifice specimen extraction surgery.

**Figure 2.**
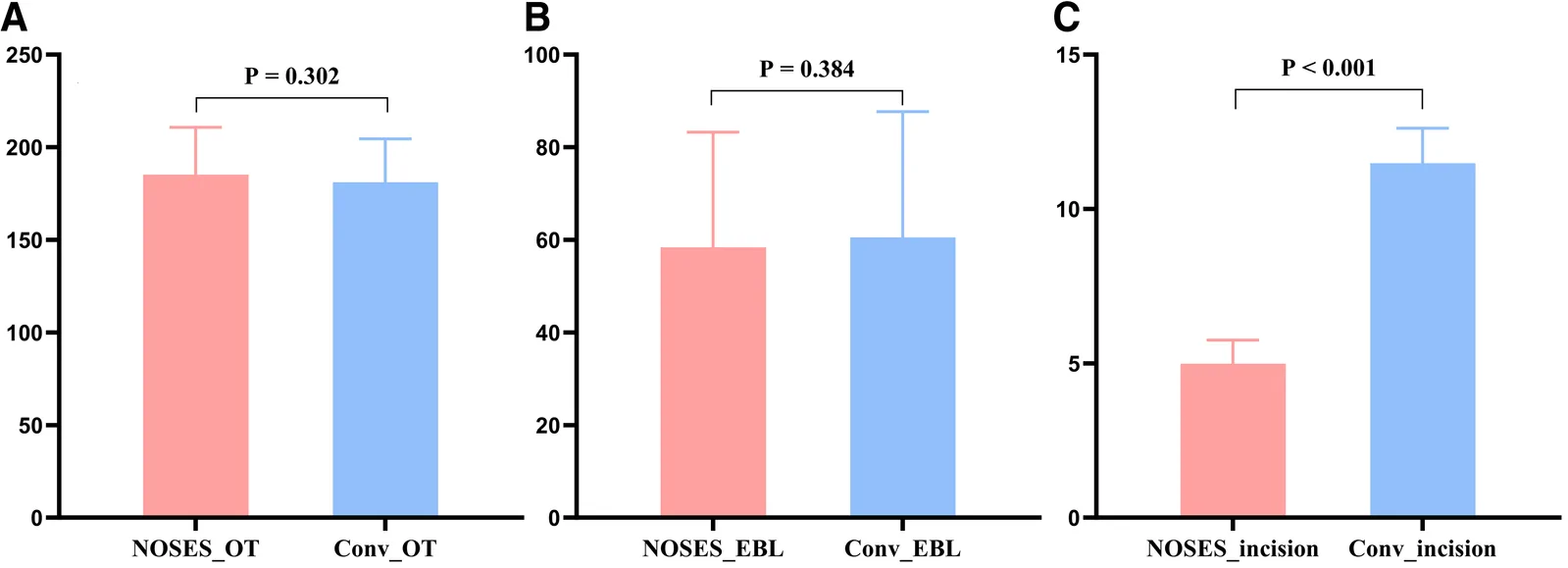
Comparison of perioperative parameters between the NOSES and conventional groups. (A) Operative time (minutes); (B) EBL (mL); and (C) length of abdominal incision (cm). Data are presented as mean ± SD. No significant differences were observed between the 2 groups for operative time or EBL (*P* > .05). The length of the abdominal incision was significantly shorter in the NOSES group compared with the conventional group (*P* < .001). EBL = estimated blood loss, NOSES = natural orifice specimen extraction surgery, SD = standard deviation.

### 3.3. Postoperative recovery

The NOSES group demonstrated significantly faster recovery across multiple parameters (Table 3). Time to first flatus was significantly shorter in the NOSES group compared with the conventional group (2.3 ± 0.9 days vs 2.9 ± 1.1 days, *P* = .002). Similarly, time to first liquid diet was significantly reduced in the NOSES group (3.1 ± 1.0 days vs 3.8 ± 1.3 days, *P* = .001). The length of postoperative hospital stay was also significantly shorter in the NOSES group (8.4 ± 3.2 days vs 10.1 ± 4.1 days, *P* = .014; Fig. 3).

**Table 3:** Postoperative recovery indicators.

Parameter	NOSES group (n = 49)	Conventional group (n = 77)	*P* value
Time to first flatus (d)	2.3 ± 0.9	2.9 ± 1.1	.002
Time to first liquid diet (d)	3.1 ± 1.0	3.8 ± 1.3	.001
Time to ambulation (d)	1.8 ± 0.7	2.1 ± 0.9	.053
Postoperative hospital stay (d)	8.4 ± 3.2	10.1 ± 4.1	.014

NOSES = natural orifice specimen extraction surgery.

**Figure 3.**
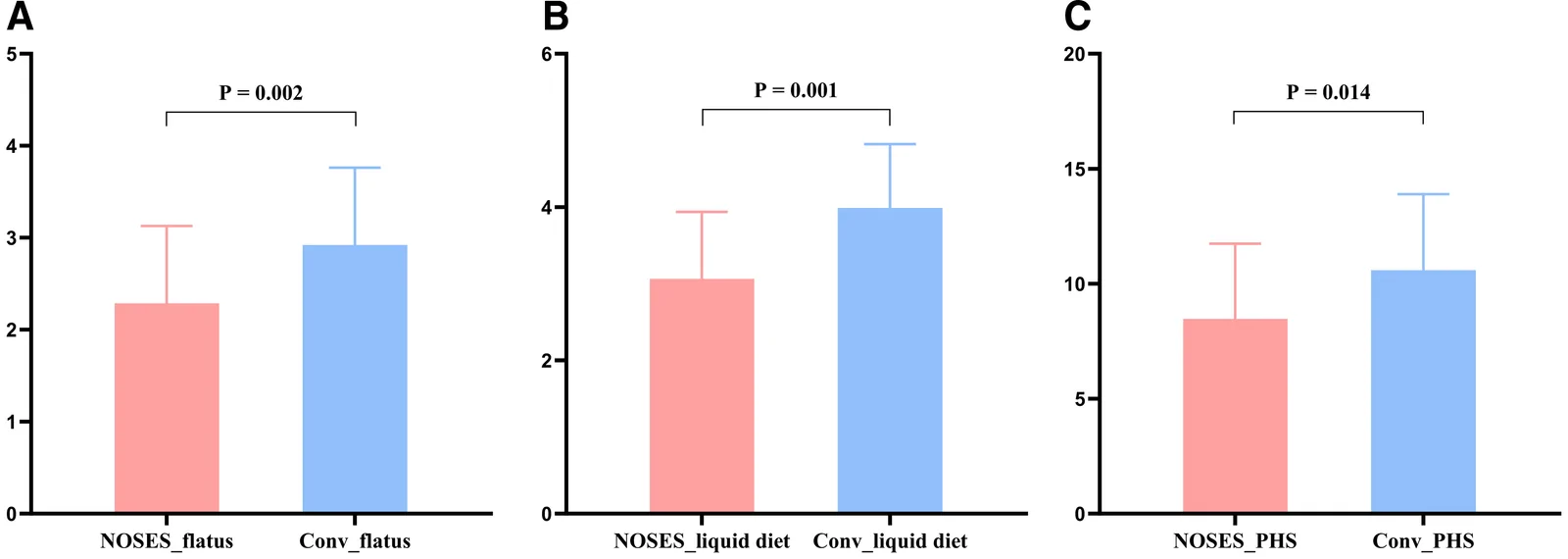
Comparison of postoperative recovery parameters between the NOSES and conventional groups. (A) Time to first flatus (days); (B) time to first liquid diet (days); and (C) postoperative hospital stay (days). Data are presented as mean ± SD. The NOSES group demonstrated significantly faster recovery across all 3 parameters compared with the conventional group. NOSES = natural orifice specimen extraction surgery, SD = standard deviation.

### 3.4. Pain assessment

Postoperative pain scores are summarized in Table 4. The NOSES group reported significantly lower VAS pain scores on POD 1 (3.2 ± 1.1 vs 4.5 ± 1.3, *P* < .001). Pain scores on POD 3 (2.1 ± 0.8 vs 2.8 ± 1.0, *P* = .082) and day 5 (1.6 ± 0.7 vs 1.9 ± 0.8, *P* = .174) also tended to be lower in the NOSES group, although the differences did not reach statistical significance. The proportion of patients requiring additional analgesics was significantly lower in the NOSES group (22.4% vs 44.2%, *P* = .014; Fig. 4).

**Table 4:** Postoperative pain assessment.

Parameter	NOSES group (n = 49)	Conventional group (n = 77)	*P* value
VAS pain score (POD 1)	3.2 ± 1.1	4.5 ± 1.3	<.001
VAS pain score (POD 3)	2.1 ± 0.8	2.8 ± 1.0	.082
VAS pain score (POD 5)	1.6 ± 0.7	1.9 ± 0.8	.174
Additional analgesics required, n (%)	11 (22.4%)	34 (44.2%)	.014

NOSES = natural orifice specimen extraction surgery, POD = postoperative day, VAS = visual analog scale.

**Figure 4.**
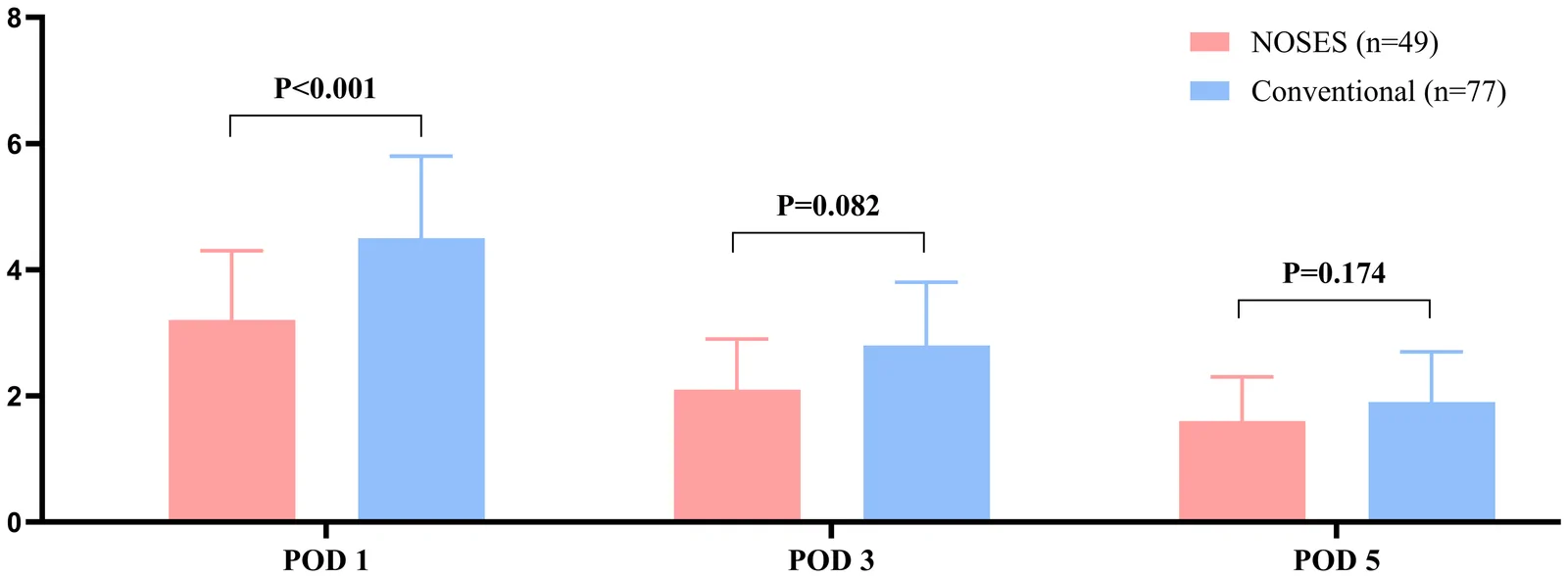
Postoperative pain scores over time. VAS scores were recorded on POD 1, 3, and 5 in the NOSES and conventional groups. Data are presented as mean ± SD. *P* < .001 for NOSES versus conventional group on POD 1. No significant differences were observed between groups on POD 3 (*P* = .082) and POD 5 (*P* = .174). The NOSES group demonstrated consistently lower pain scores throughout the early postoperative period, with the greatest difference observed on the first postoperative day. NOSES = natural orifice specimen extraction surgery, POD = postoperative day, SD = standard deviation, VAS = visual analog scale.

### 3.5. Postoperative complications

The incidence of postoperative complications is presented in Table 5. The overall complication rate was lower in the NOSES group compared with the conventional group (12.2% vs 24.7%), with an absolute risk difference (ARD) of −12.5% (95% confidence interval [CI] = −24.2% to −0.8%, *P* = .043 by Fisher exact test). Notably, wound-related complications – including surgical site infection and wound dehiscence – occurred exclusively in the conventional group (0% vs 6.5%; ARD = −6.5% [95% CI = −12.9% to −0.1%], *P* = .045 by Fisher exact test). Given the small number of events (0 vs 5), however, this finding should be interpreted with appropriate caution and is best considered suggestive of a potential benefit rather than conclusive evidence. No significant differences were observed between groups regarding anastomotic leakage (4.1% vs 5.2%; ARD = −1.1% [95% CI = −8.7% to 6.5%], *P* = .775), intra-abdominal abscess (2.0% vs 3.9%; ARD = −1.9% [95% CI = −8.3% to 4.5%], *P* = .569), ileus (4.1% vs 3.9%; ARD = 0.2% [95% CI = −7.2% to 7.6%], *P* = .956), urinary retention (0% vs 2.6%; ARD = −2.6% [95% CI = −7.6% to 2.4%], *P* = .262), or pulmonary infection (2.0% vs 2.6%; ARD = −0.6% [95% CI = −6.6% to 5.4%], *P* = .838). With respect to complication severity, the incidence of Clavien-Dindo grade I/II complications was 10.2% in the NOSES group versus 20.8% in the conventional group (ARD = −10.6% [95% CI = −22.4% to 1.2%], *P* = .124), while grade III complications occurred in 2.0% and 3.9%, respectively (ARD = −1.9% [95% CI = −8.3% to 4.5%], *P* = .569). All *P* values reported for complication comparisons were derived from Fisher exact test.

**Table 5:** Postoperative complications with absolute risk differences and 95% CIs.

Complication	NOSES group (n = 49)	Conventional group (n = 77)	Absolute risk difference (95% CI)	*P* value
Overall complications, n (%)	6 (12.2)	19 (24.7)	−12.5% (−24.2% to −0.8%)	.043
Wound infection, n (%)	0 (0)	5 (6.5)	−6.5% (−12.9% to −0.1%)	.045
Anastomotic leakage, n (%)	2 (4.1)	4 (5.2)	−1.1% (−8.7% to 6.5%)	.775
Intra-abdominal abscess, n (%)	1 (2.0)	3 (3.9)	−1.9% (−8.3% to 4.5%)	.569
Ileus, n (%)	2 (4.1)	3 (3.9)	0.2% (−7.2% to 7.6%)	.956
Urinary retention, n (%)	0 (0)	2 (2.6)	−2.6% (−7.6% to 2.4%)	.262
Pulmonary infection, n (%)	1 (2.0)	2 (2.6)	−0.6% (−6.6% to 5.4%)	.838
Clavien-Dindo grade I/II, n (%)	5 (10.2)	16 (20.8)	−10.6% (−22.4% to 1.2%)	.124
Clavien-Dindo grade III, n (%)	1 (2.0)	3 (3.9)	−1.9% (−8.3% to 4.5%)	.569

Data are presented as number (percentage). Absolute risk differences were calculated as the rate in the NOSES group minus the rate in the conventional group; negative values favor the NOSES approach. All *P* values were derived from Fisher exact test. Given the limited event counts, 95% CIs for absolute risk differences and the associated *P* values should be interpreted with appropriate caution.

CI = confidence interval, NOSES = natural orifice specimen extraction surgery.

### 3.6. Pathological outcomes

Pathological outcomes are summarized in Table 6. The mean number of harvested lymph nodes was comparable between the NOSES and conventional groups (16.3 ± 4.2 vs 15.7 ± 3.9, *P* = .414), and both groups exceeded the recommended minimum of 12 lymph nodes. The positive resection margin rate was 0% in both groups (R0 resection achieved in all patients). No significant differences were observed regarding tumor diameter, histological differentiation, or pathological stage distribution.

**Table 6:** Pathological outcomes.

Parameter	NOSES group (n = 49)	Conventional group (n = 77)	*P* value
Tumor diameter (cm)	3.1 ± 1.3	3.3 ± 1.5	.437
Harvested lymph nodes (n)	16.3 ± 4.2	15.7 ± 3.9	.414
Positive lymph nodes (n)	1.4 ± 2.1	1.6 ± 2.3	.618
R0 resection, n (%)	49 (100%)	77 (100%)	1.000
Histological differentiation (well/moderate/poor)	8/31/10	14/47/16	.886

NOSES = natural orifice specimen extraction surgery.

## 4. Discussion

This single-center retrospective study demonstrates that NOSES offers distinct advantages over conventional minilaparotomy-assisted specimen extraction in laparoscopic CRC resection, while maintaining comparable short-term oncological integrity.

The significantly shorter time to first flatus and earlier resumption of oral intake in the NOSES group are consistent with published evidence. A systematic review and meta-analysis of 28 studies involving 3247 subjects demonstrated that vaginal natural orifice transluminal endoscopic surgery was associated with significantly shorter hospital stays than laparoscopy (mean difference = 0.59, 95% CI = 0.32–0.89) and lower postoperative complication rates (odds ratio = 0.54, 95% CI = 0.23–1.0).^[16]^ These findings corroborate that eliminating abdominal incisions for specimen extraction confers meaningful advantages in postoperative recovery, likely resulting from reduced bowel manipulation and avoidance of bowel exteriorization.^[17]^

The significantly lower VAS pain scores on POD 1 and reduced analgesic requirements in the NOSES group align with previous meta-analyses reporting improved pain scores (mean difference = −1.06, 95% CI = −3.74 to −0.37).^[18]^ The mechanism is likely multifactorial, encompassing the absence of an abdominal extraction incision, reduced wound tension, and diminished nociceptive stimulation. The convergence of pain scores by POD 5 suggests that the analgesic advantage of NOSES is most pronounced in the early postoperative period.^[19]^ Nevertheless, the lack of a strictly standardized analgesic protocol represents a limitation inherent to the retrospective design; individual prescribing decisions remained at the discretion of the attending surgeon, which may have influenced VAS scores and analgesic requirements. The consistent magnitude and direction of the pain-related findings across multiple measures, however, lend support to the observed benefit. Regarding postoperative hospital stay, this parameter is subject to institutional discharge policies and nonclinical factors; accordingly, this finding should be interpreted as a descriptive indicator rather than definitive evidence of superior recovery.

The reduction in wound-related complications represents one of the most compelling arguments for NOSES. In the present study, wound infections occurred exclusively in the conventional group (0% vs 6.5%, *P* = .045), consistent with the meta-analysis by Wang et al reporting an odds ratio of 0.15 (95% CI = 0.07–0.31) for incision-related complications favoring NOSES.^[12]^ The overall complication rate in the NOSES group (12.2%) was lower than that in the conventional group (24.7%, *P* = .043), corroborating cumulative evidence demonstrating reduced overall postoperative morbidity with NOSES. Of note, the borderline *P* value and the wide CI for the ARD – which approaches zero – underscore the need for cautious interpretation.

Despite theoretical concerns regarding peritoneal contamination, no significant difference in intra-abdominal abscess rates was observed between groups (2.0% vs 3.9%, *P* = .569), consistent with previous meta-analyses demonstrating that NOSES does not increase postoperative infectious complications.^[20]^ The comparable anastomotic leakage rates (4.1% vs 5.2%, *P* = .775) align with multiple meta-analyses suggesting that transanal specimen extraction and intracorporeal anastomosis do not compromise anastomotic integrity when performed with appropriate technique.^[21,22]^

The oncological safety of NOSES warrants careful interpretation. In the present study, harvested lymph nodes were comparable between groups (16.3 ± 4.2 vs 15.7 ± 3.9, *P* = .414), and R0 resection was achieved in all patients. These findings are reassuring but insufficient to establish oncological equivalence. Although a meta-analysis of 16 studies found comparable 3-year disease-free and overall survival between NOSES and conventional laparoscopy groups,^[23]^ the present study lacks long-term follow-up, recurrence data, disease-free survival, or peritoneal/tumor-cell contamination information. Consequently, our conclusions regarding oncological safety must be strictly confined to short-term perioperative outcomes and pathological surrogate measures – namely lymph node yield and R0 resection status – rather than extended to long-term oncological efficacy.

The comparable operative time between groups (186.4 ± 42.7 minutes vs 178.9 ± 38.5 minutes, *P* = .302) reflects the accumulated experience of our surgical team; however, the learning curve associated with intracorporeal anastomosis and transanal extraction has been well-documented and may have influenced outcomes in the earlier phase of the study period, introducing potential calendar-time bias.^[24]^ Furthermore, while our surgical team adhered to standardized oncological principles – including correct vascular control and mesocolic/mesorectal plane dissection – these technical fundamentals remain central to successful outcomes, and the benefits of specimen extraction route should not overshadow the importance of proper anatomical technique.^[25]^ Accurate preoperative imaging and localization^[26]^ are also critical for appropriate patient selection for NOSES, particularly in assessing tumor size, local stage, and circumferential resection margin risk.

Several limitations warrant acknowledgment. The retrospective design inherently predisposes to selection bias, notwithstanding the satisfactory covariate balance demonstrated by standardized differences (all < 0.1). We acknowledge the absence of propensity score matching or multivariable regression adjustment; however, the limited sample size and paucity of outcome events precluded stable multivariate modeling due to overfitting risks. The modest sample size (126 patients) further limits statistical power for infrequent complications and precludes meaningful subgroup dissection. The single-center design restricts external validity, while the absence of long-term surveillance precludes assessment of disease-free or overall survival. The stringent eligibility criteria – BMI < 30 kg/m^2^, tumor diameter ≤ 5 cm, exclusion of neoadjuvant therapy, emergency presentations, metastatic disease, and non-sigmoid/rectal subsites – were deliberately instituted to ensure technical safety and procedural feasibility, yet they substantially circumscribe generalizability to unselected CRC populations. Even though tumor location did not reach statistical significance between groups, the numerical imbalance (59.2% vs 50.6% for sigmoid tumors) may still have introduced subtle confounding. In addition, the absence of an enhanced recovery after surgery protocol – which was not uniformly implemented during the study period – may have influenced postoperative recovery parameters independent of the surgical technique. Patient-reported outcomes, including cosmetic satisfaction and quality-of-life measures, were not systematically captured, and cost-effectiveness data were also unavailable, representing critical avenues for future prospective investigation.

Despite these limitations, the present study adds to the growing evidence supporting NOSES as a safe and effective alternative to conventional minilaparotomy-assisted specimen extraction in selected patients with CRC. The benefits in postoperative recovery, pain control, and wound-related complication reduction are clinically meaningful. Nevertheless, given the single-center nature and the highly selected cohort, these findings require validation in larger, multicenter prospective studies with extended follow-up before definitive conclusions regarding broad applicability and long-term oncological safety can be drawn.

## 5. Conclusions

NOSES appears to be a safe and feasible surgical approach for specimen extraction in laparoscopic CRC resection. Compared with conventional minilaparotomy-assisted extraction, NOSES offers significant advantages in postoperative pain control, gastrointestinal function recovery, and wound-related complication reduction, without compromising oncological integrity as reflected by comparable lymph node yield and R0 resection rates. The elimination of the abdominal extraction incision effectively reduces wound-related morbidity while preserving the oncological principles of radical resection. These findings support the broader application of NOSES only in appropriately and highly selected patients with CRC, and extrapolation to unselected populations should be undertaken with caution. Nevertheless, further prospective randomized controlled trials with larger sample sizes and extended follow-up are warranted to confirm these benefits and establish the long-term oncological safety of this technique.

## Author contributions

**Conceptualization:** Yongjian Liao.

**Data curation:** Yongjian Liao, Runfeng Qiu.

**Formal analysis:** Yongjian Liao, Runfeng Qiu.

**Funding acquisition:** Yongjian Liao.

**Investigation:** Yongjian Liao, Wenli Ma.

**Methodology:** Yongjian Liao, Runfeng Qiu.

**Resources:** Runfeng Qiu.

**Writing – original draft:** Yongjian Liao, Wenli Ma.

**Writing – review & editing:** Yongjian Liao, Runfeng Qiu, Wenli Ma.
